# A Quick Guide for Computer-Assisted Instruction in Computational Biology and Bioinformatics

**DOI:** 10.1371/journal.pcbi.1000035

**Published:** 2008-04-25

**Authors:** Manuel João Costa, Eduardo Galembeck, Guilherme A. Marson, Bayardo B. Torres

**Affiliations:** 1 Life and Health Sciences Research Institute (ICVS), School of Health Sciences, University of Minho, Gualtar Campus, Braga, Portugal; 2 Biochemistry Department, Institute of Biology, University of Campinas, Campinas, Brazil; 3 Health Science e-Training Foundation, Institute Suisse de Recherche Expérimentale sur le Cancer, Epalinges sur Lausanne, Switzerland; 4 Biochemistry Department, University of São Paulo, São Paulo, Brazil; Whitehead Institute, United States of America

## Introduction

Computational Biology and Bioinformatics (CBB) are indispensable components in the training of life scientists [1]–[3]. Current curricula in the life sciences should prepare graduates who master quantitative and computer skills for increased levels of performance [4]–[6]. Equally important is that the application of the curricula is driven by an appropriate instructional paradigm and effective learning experiences. Teaching and learning with computers bring specific issues that should be considered beforehand by any instructor. The following Quick Guide for Computer-Assisted Instruction (CAI) outlines ten principles for effective teaching. The principles are aligned with current developments on human cognition and learning [7] and have been drawn from our own experience using CAI in seminars, tutorials, and distance education, in courses on Molecular Life Sciences at the undergraduate level, taught to majors in biology or in other subjects (e.g., nutrition, teaching of physics and chemistry, teaching of biology, sports).

The Guide refers to the preparation, presentation, and assessment of CAI. It should be an aid for those who teach CBB with CAI in class, and it is expected to stimulate student motivation and deeper learning in CBB, thus making class time more effective and improving satisfaction of both students and instructors.

## 1. Ensure That CAI Activities Are Integrated into Your Curriculum

CAI activities in a course should not be isolated exercises, but should be embedded in lesson plans and integral to the instructor's goals [8]. The instructor should be very explicit about what students are expected to achieve with computer activities (see principle 7). For example, to ignite student interest on metabolism at the systems level, simulations of metabolic conditions associated to sports have been proven quintessential, even when simulations are used in lecture halls [9]. Also, activities have been reported in which CAI is contextualized by problems that require the of mastering CBB [10]. Explicit statements on CAI should be included in the complete list of instructional objectives, and should be carefully defined in terms of both the content and the skills to be addressed [5]. The use of software should take into consideration student computational and visual skills so that they can make the most of CAI sessions.

## 2. Do Not Overuse CAI

CAI is the first option, if the goal is developing students' IT skills or other skills difficult to attain in the real world. For instance, computer-based laboratory simulations have been used in place of dangerous, time-consuming, ethically constrained, or expensive experiments [11],[12]. However, for the majority of instructional objectives, CAI is one among several alternative teaching strategies. If a strategy currently in use is effective, do not replace it automatically with CAI [13]. Analyze advantages and drawbacks of CAI, and let the results of the analysis dictate the decision. For example, software that simulate chromatography will always be precious learning tools, but students who are taking an advanced course on biochemistry might need to go through real laboratory classes. A well-balanced repertoire of instructional approaches is a major characteristic of successful teaching [14]. For instance, effective blended approaches in which pencil and paper activities are articulated with CAI have been reported in introductory CBB courses [5].

**Figure 1:**
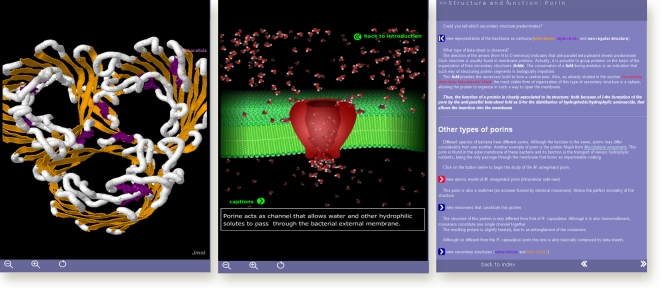
Tutorial with molecular visualization. Screenshot of a tutorial with step-by-step instructions (right) that combine 3-D visualization (left) of the structure of porine with a 2-D (center) animation which illustrates its biological role. (Source: tutorial by Marson GA, et al. Available: http://www.iq.usp.br/wwwdocentes/bayardo/softwares/english/studyprot/menu/mainMenu.html. Accessed 2 April 2008.).

## 3. Plan for Uses of CAI Adjusted to Infrastructure and Resources Available

Inadequate infrastructure and deficient on-site technical or teaching assistance can limit the effectiveness of CAI applications, so plans should be adjusted to existing conditions.

The following items should be included in a checklist: (a) facilities (physical space, number of machines, etc.); (b) characteristics of the computers (CPU performance, display size, and resolution, etc.); (c) technical support (essential for setup and troubleshooting); (d) onsite teaching assistants; (f) ethical and copyright issues; (g) connectivity and network. All instructors should be familiar with the CAI resources. Therefore, training sessions should be provided for instructors when necessary.

## 4. Maximize Interactivity

Science and engineering students are more motivated and learn better when they are actively engaged than when they are simply watching and listening [15],[16]. Give preference to computer resources that provide engagement [17]. Effective applications often require students to make decisions through a context-sensitive system. Further examples include simulators of biological processes [18] and tutorials based on incremental cycles of data presentation, user action, and system feedback (Figure 1). Notice that while a tutorial provides a pathway for the learner, a simulator does not (as a consequence, many learners require external orientation). Tutorials also provide more control over the duration and the products of instruction [19]–[21]. Some ingenious applications combine both approaches by embedding a simulator into a tutorial [22]. When software does not include a tutorial, the definition of appropriate exploratory pathways rests with the instructional design. For instance, research papers have been converted into case studies which required students to use online resources to explore sequences or structures [22]. Whatever your choice, minimize the need for screen reading of both text and diagrams.

## 5. Allow for Different Rates of Progression in Class, but Ensure That All Students Reach the Objectives

Student-centered learning in class can be implemented with CAI [7],[8],[23]. Once the instructional objectives are explicit and available to students (see principle 7), you should allow variations in individual or team progression in the same class without considering it a disadvantage for students or a threat to your control. In fact, it is quite the opposite: different paces stimulate peer collaboration and classes become easier to manage [24]. Another advantage is the stimulus to the development of the ability to communicate concepts [24]. In this regard, grouping students from biology and computer science backgrounds has been reported to be a rich exchange opportunity to sum up complementary competencies in bioinformatics classes [25]. A CAI class in which all the students follow the rhythm of the instructor could be a lecture in disguise.

## 6. Define Milestones and Coach Students through Them

Providing the appropriate guidance becomes critical when CAI is used with complex problems [26]. Students should not be too lost, nor should they be guided to the extent that they become mentally inactive. Milestones or checkpoints for the achievement of certain goals can both facilitate class progress and allow it to be monitored. For example, we have observed that there may be a number of students who do not pay too much attention to an activity that is simply recommended and never checked explicitly. In our experience, defining beforehand the evidence that will be asked for from every student and requesting that evidence in a timely manner can put students back on a good track. Therefore, define milestones for roughly every 20 minutes and use them as checkpoints; stop the class periodically and give more direct guidance to anyone who needs it. A good way of committing students to checkpoints is to assign credits to those who make appropriate progress. Coach them; emphasize successes, and encourage learning from failures.

## 7. Ensure Students Understand the Scope and Objectives of Assignments

Make sure that your students read and understand the CAI tasks, the deadlines, and their role in instruction. Present instructional objectives in terms of contents and skills, for example, “at the conclusion of this exercise you should be able to search a database for specific gene sequences.” Adjust the intended conceptual depth and mastery of skills to a feasible level. A good challenge pulls the student ahead and promotes learning, whereas objectives that are out of reach result in frustration. Keep in mind that students often find it difficult to work to achieve instructional objectives. A clear understanding of goals will increase student motivation, independence, and satisfaction with the CAI class [27].

## 8. Be Sure Students Understand the Models Presented on the Screen

The dynamic presentation of processes and theoretical models is a great strength of CAI [28]. When teaching with models, pay special attention to the following issues: (a) students have different levels of visual literacy, thus they might interpret and understand visuals (animated or static) in very different ways [29]; (b) models represent reality but are not reality, so students should understand what a model can and cannot explain; (c) students often interpret models according to previous misconceptions, which can seriously impair learning [29]. Address these issues by communicating with your students before and during CAI. Pose questions about the models that require more than rote memorization. Finally, choose the models based on clarity, accuracy, and adequate representation. Stunning but overly busy animations may transform your CAI into mere entertainment.

## 9. Assess and Evaluate Student Performance When You Use CAI

Always be aware that assessment drives learning. Students tend to ignore instructional activities that make no contributions to marks [30]. It is therefore of the utmost importance that you assess at least some of the CAI outcomes. This means examining and marking students for CAI performance, which may be done through written tests (cognitive interpretations) or computer exercises. For example, in CAI of statistics, we have observed that including the actual performance in the use of the software as an assessment item resulted in higher learner commitments. Campbell describes Web-based assignments which target student knowledge on CBB, mastering of online Bioinformatics tools, and the most complex cognitive levels [23]. In your course notes, be very clear that you will do the assessment, and provide the essential information regarding when and how you will do it. Answers to evaluation questions embedded in instructional software can be considered for evaluation purposes [9],[31].

## 10. Use the Computer under an Appropriate Paradigm

CAI is not the only solution in education, and your syllabus may be better taught by alternative methodologies. CAI is powerful in achieving educational goals such as the development of skills involved in data searching, integration and analysis, leverage of IT proficiency (in both synchronous and asynchronous modalities), and the development of visual literacy. However, merely using keyboards and screens instead of pen and paper does not guarantee improvements in teaching or learning [32]. If you are considering adopting CAI, focus on the paradigm in which you will use it.
